# Roles of Lipolytic enzymes in *Mycobacterium tuberculosis* pathogenesis

**DOI:** 10.3389/fmicb.2024.1329715

**Published:** 2024-01-29

**Authors:** Hong Lin, Jiayin Xing, Hui Wang, Shuxian Wang, Ren Fang, Xiaotian Li, Zhaoli Li, Ningning Song

**Affiliations:** ^1^Weifang Key Laboratory of Respiratory Tract Pathogens and Drug Therapy, School of Life Science and Technology, Shandong Second Medical University, Weifang, China; ^2^SAFE Pharmaceutical Technology Co. Ltd., Beijing, China

**Keywords:** *Mycobacterium tuberculosis*, lipolytic enzymes, virulence factor, pathogenicity, therapeutic targets

## Abstract

*Mycobacterium tuberculosis* (Mtb) is a bacterial pathogen that can endure for long periods in an infected patient, without causing disease. There are a number of virulence factors that increase its ability to invade the host. One of these factors is lipolytic enzymes, which play an important role in the pathogenic mechanism of Mtb. Bacterial lipolytic enzymes hydrolyze lipids in host cells, thereby releasing free fatty acids that are used as energy sources and building blocks for the synthesis of cell envelopes, in addition to regulating host immune responses. This review summarizes the relevant recent studies that used *in vitro* and *in vivo* models of infection, with particular emphasis on the virulence profile of lipolytic enzymes in Mtb. A better understanding of these enzymes will aid the development of new treatment strategies for TB. The recent work done that explored mycobacterial lipolytic enzymes and their involvement in virulence and pathogenicity was highlighted in this study. Lipolytic enzymes are expected to control Mtb and other intracellular pathogenic bacteria by targeting lipid metabolism. They are also potential candidates for the development of novel therapeutic agents.

## 1 Introduction

Tuberculosis (TB), which is caused by infection with *Mycobacterium tuberculosis* (Mtb), is identified as the one of the earliest human illnesses that continue to be among the most lethal infectious diseases. Tuberculosis (TB) was the second most common infectious illness killer globally after COVID-19 in 2022. It was also the primary cause of death for those who tested positive for HIV and a major factor in deaths linked to antibiotic resistance. In 2022, there were 10.6 million cases of tuberculosis reported globally. Between 2020 and 2022, the incidence rate of tuberculosis (TB) climbed by 3.9% per 100,000 people annually, reversing a two-decade trend of annual declines of about 2% (WHO, 2023). Despite the various interventions that have been used to prevent and treat TB, the cure for the disease is yet to be found. The main reason for this is that Mtb is an incredibly complex and unique pathogen that can evade the immune system (Bullen et al., 2023; Sengupta et al., 2023; Shariq et al., 2023). The primary form of TB is usually found in macrophages, where the bacteria can survive and replicate (Dey and Bishai, 2014). However, the exact pathogenicity mechanism remains unclear (Li et al., 2019).

To treat this disease and approach a world free of tuberculosis, new vaccines and medications should take Mtb virulence characteristics into account. Studies on the molecular mechanisms underlying the pathogenicity, virulence, and persistence of mycobacteria made significant strides in recent years. The discovery of crucial mycobacterial virulence genes has been one of the noteworthy achievements. The majority of these virulence genes encode regulators, cell surface proteins, lipid pathway enzymes, and proteins that are involved in signal transmission.

This review focuses on Mtb lipolytic enzymes whose inactivation results in a significant reduction in the levels of pathogenicity or virulence. Forrellad et al. (2013) reported that the virulence determinants were categorized into the following groups based on their function. We ranked them in order of relevance to the role of lipolytic enzymes: (1) lipid and fatty acid metabolism, including the catabolism of cholesterol; (2) macrophage-inhibiting proteins, such as those involved in response to nitrosative and oxidative stress, phagosome arresting, and inhibition of apoptosis; (3) cell envelope proteins, such as lipoproteins, cell wall proteins, and secretion systems; (4) proteases, including metalloproteases; (5) protein kinases; (6) importer and exporter proteins for metal transport; (7) proteins with unknown functions, such as the PE and PE-PGRS families, PE are distinguished by around 100 amino acid conserved N-terminal domains (Cole et al., 1998). The characteristic motif Pro (P) - Glu (E) is where the name PE originates. The largest subfamily of PE is called PE-PGRS, and it is distinguished by PE N-terminal domain and PGRS (Polymorphic GC-rich Repetitive Sequences) in C-terminal domain; (8) transcriptional regulators, such as sigma factors and two component systems; (9) additional virulence pr oteins.

Mtb differs from pathogenic bacteria in that it has a wide range of intricate lipids and lipoglycans on its cell envelope. From the inside out, the components of the Mtb cell envelope are as follows: (i) plasma membrane; (ii) cell wall made up of different non-covalently linked proteins, lipids and carbohydrates, as well as three covalently linked macromolecules (mycolic acids, arabinogalactan and peptidoglycan) and (iii) a capsule composed of lipids, proteins and polysaccharides (Brennan, 2003). Pathogenic mycobacteria are distinguished by their unique cell envelope, which comprises various lipids esterified with structurally related long-chain multi-methyl-branched fatty acids. It has long been believed that these lipids are crucial for both the virulence and structure of the tubercle bacillus cell envelope. It is known that Mtb has 250 kinds of genes encoding putative enzymes involved in lipid metabolism from *in silico* analysis (Cole et al., 1998), while only 50 such genes encoding for these enzymes in *Escherichia coli*, which has a similar genome size (Neyrolles and Guilhot, 2011). The lipolytic enzymes involved in lipid metabolism are mainly hydrolyzing carboxyl ester to fatty acids and glycerol, which are utilized in colonization, persistence, virulence and as an energy source. Based on the specificity of their respective substrates, enzymes related to lipid metabolism can be divided into four main categories: esterases, lipases, phospholipases, and cutinases, detailed information on these enzymes are below.

## 2 The classification of lipolytic enzymes in *Mycobacterium tuberculosis*

Lipolytic enzymes such as lipases, esterases, phosphlipases, and cutinases are significantly involved in the pathological processes that enhance their survival. However, more of their characteristics still need to be studied in detail. Studies on mycobacteria infection revealed the role played by lipolytic enzymes in pathogenicity. Lipolytic enzymes share the GXSXG pentapeptide sequence, which is a feature of the α/β hydrolase fold family of proteins (Johnson, 2017). In general, four types of lipolytic enzymes can be identified, based on the characteristics and degree of specificity of the relevant substrates (Delorme et al., 2012; Dedieu et al., 2013). These four classes of lipolytic enzymes include: (1) lipases, with the consensus sequence GXSXG, which hydrolyze water-insoluble long-chain carboxylesters like long-chain triglycerides (TAG); (2) esterases (or carboxylesterases), with the consensus sequence GXSXG, which hydrolyze small and partially water-soluble carboxylesters; (3) phospholipases, with the consensus sequence G-X_1_-S-X_2_-G, which are sub-classified into four groups (PLA1, PLA2, PLC and PLD) cleaving the different bond position of phospholipids; (4) cutinases, with the consensus sequence G-[YF]-S-[QL]-G, which break down all types of carboxylesters, including TAG, and phospholipids, as well as cutin. Cole et al. (1998) annotated 24 putative lipase/esterase gene from the Mtb genome, known as the “Lip family.” However this classification does not differentiate between lipases and esterases. This review is mainly introduced the lipolytic enzymes in the following four categories: Lip family, other lipase/esterase, phospholipases and cutinases. Creating bioactive compounds and substrates that serve as the carbon and energy sources depend on bacterial lipolytic enzymes. Additionally, the bacterial lipolytic enzymes play a significant role in controlling the host’s protective immunological responses and signal transduction cascades. Due to space limitation, Table 1 only summarizes some representative well-characterized lipolytic enzymes that are currently known. Others are in Supplementary Table S1.

**Table 1:** Lipolytic enzymes of *M. tuberculosis* H37Rv and their function.

Enzyme classification	Gene Product	Subcellular localization	Enzymatic activity	Function	References
Lip family	LipX (PE11, Rv1169c)	**Cell wall	**PE family **Esterase **Hydrolysis of *p-*NP acetate Specific activities: 1215 mU mg^−1^ with Tween 20	**LipX modifies lipid content and cell wall architecture **Stimulates cytokines like IL-10 and IL-4 to create an environment that is primarily of the Th2-type **Mtb ΔLipX had poorer survival in activated THP-1 macrophages	Cascioferro et al. (2007), Deng et al. (2015), Singh et al. (2016), Rastogi et al. (2017)
	LipY (Rv3097c)	**Cytoplasmic **Cell envelop	**Lipase **Hydrolysis of TAG **PE-PGRS family Specific activities: 41 nmol mg^−1^ min^−1^ with triolein *K*_*m*_: 7.57 mmol L^−1^ and *V*_*max*_ of 653.3 nmol mg^−1^ min^−1^	**LipY inhibits Th1 and Th17 responses and stimulates Treg cell induction **LipY participates in the metabolism of lipids	Deb et al. (2006), Mishra et al. (2008), Daleke et al. (2011), and Singh et al. (2014)
Other esterase/lipase	Rv2224c (MT2282; Hip1; CaeA)	**Cell wall	**Serine protease **Carboxyesterase	**Reduces proinflammatory response, inhibits antigen presentation and T cell responses **It is required for virulence of Mtb	Lun and Bishai (2007), Rengarajan et al. (2008), Madan-Lala et al. (2011, 2014), and Naffin-Olivos et al. (2014)
	Rv0183	**Cell wall	**Lipase **Hydrolysis of MAG **Phospholipase Specific activities: 27 U mg^−1^	*Possible function in the lipid metabolism of the host cell membrane **Alveolar macrophages involved in tuberculosis physiology exhibit inflammatory markers as IL-6, NF-_k_B, TLR2, TLR6, TNF-ɑ and MyD88 when Rv0183 is present	Côtes et al. (2007), Xu et al. (2010), and Liu et al. (2018)
Phospholipase	PLC	**Cell wall **Membrane	**Phospholipases **Hydrolysis of *p*-NP choline (*p*-NPC) Specific activities (μ mol min^−1^ mg^−1^): PLC-A: 10.5, PLC-B: 10.1, PLC-C: 9.3, PLC-D: 9	**Help mycobacteria adapt to the iron-limited intracellular environment **Hydrolytic activity on the phospholipids in the host cell membrane **PLC-encoding genes were strongly upregulated under phosphate starvation	Bacon et al. (2007), Bakala N’goma et al. (2010), and Le Chevalier et al. (2015)
Cutinase	Culp6/ Cut6 (Rv3802c)	**Cell wall	**Phospholipase A **Thioesterase **Lipase With *p*-NPB Specific activity: 40 pmol min^−1^ mg^−1^ *V_max:_* 1.62 mol min^−1^ mg^−1^ *k_cat_*: 8.81 S^−1^ *K_m:_* 23.52 mmol L^−1^	**Cut6 participates in the production of mycolic acid **Essential for *in vitro* growth of the bacilli **Cut6 promotes the production of IFN-γ by Th1-type T cells	Sassetti et al. (2003), Mattow et al. (2007), West et al. (2008), 2009, Parker et al. (2009), Crellin et al. (2010), and Shanahan et al. (2010)

*Bioinformatics prediction.

**Experimentally defined.

### 2.1 Lip family

From the genome annotation, it has been shown that 24 genes (C to Z, excluding A and B) may encode lipolytic enzymes known as “Lip family” (Table 1 and Supplementary Table S1). The consensus sequence GXSXG, which is a feature of members of the α/β hydrolase fold family, is the only factor used to categorize these proteins. This classification does not differentiate between lipases and esterases. Genome sequence analysis cannot be used as the single criterion for categorizing the proteins. The only method for differentiating between lipase and esterase enzymes is based on biochemical characterization. Lipases hydrolyze water-insoluble long-chain carboxylesters like TAG, while esterases hydrolyze small and partially water-soluble carboxylesterases. The “Lip family” is made up of both lipase and esterase enzymes (Cole et al., 1998; Camus et al., 2002; Canaan et al., 2004; Delorme et al., 2012; Shen et al., 2012; Li et al., 2017; Yang et al., 2019). The LipC (Rv0220) (Shen et al., 2012), LipD (Rv1923) (Singh et al., 2014), LipE (Rv3775) (Yang et al., 2019), LipF (Rv3487c) (Delorme et al., 2012), LipH (Rv1399c) (Canaan et al., 2004), LipJ (Rv1900c) (Kumari and Kaur, 2021), LipK (Rv2385) (Chownk et al., 2018), LipL (Rv1497) (Dey et al., 2022), LipN (Rv2970c) (Jadeja et al., 2016), LipR (Rv3084) (Zhang et al., 2019), LipS (Rv3176c) (Chownk et al., 2017), LipU (Rv1076) (Li et al., 2017), LipW (Rv0217c) (Delorme et al., 2012), and LipX (Rv1169c) (Singh et al., 2016) are functionally characterized as esterases. LipD (Rv1923) (Singh et al., 2014), LipQ (Rv2485c) (Kumar et al., 2017a), LipT (Rv2045c) (Singh et al., 2010), and LipY (Rv3097c) (Singh et al., 2014) are functionally characterized as lipases. Among 24 lipolytic enzymes in Lip family, there are 12 proteins (LipC, LipF, LipH, LipI, LipM, LipN, LipO, LipQ, LipR, LipU, LipW, LipY) homologous to the human Hormone Sensitive Lipase (hHSL). The epinephrine-sensitive lipolytic enzyme hormone-sensitive lipase (HSL) was originally discovered in adipose tissue (Vaughan et al., 1964). This enzyme family, also known as the “Lip-HSL” family, is essential for the release of free fatty acid from TAG that is kept in adipocytes (Lafontan and Langin, 2009; Lampidonis et al., 2011). The conserved GXSXG and HGGG motifs, which include the catalytic serine and oxyanion hole, respectively, are present in the core α/β hydrolase domain of HSL.

### 2.2 Other lipases/esterases

In recent years, more enzymes have been identified as lipases/esterases through experiments. A series of enzymes containing Rv3091, Rv0183, Rv1592c, Rv2037c, and Rv1683 are functionally characterized as lipases. Rv0774c, Rv1075c, Rv3036c, Rv0045c, Rv1430, and Rv3539 are functionally characterized as esterases. Rv2224c and Rv0519c, with the catalytic triad that is found in esterases, lipases, and proteases, are identified as lipases/esterases by experiments (Ferre and Clote, 2005; Srivastava et al., 2008).

With the development of bioinformatics, comparative proteomics studies are increasingly being undertaken to find new virulence factors, like therapeutic targets and vaccine candidates (Cole et al., 1998). Nearly 40% of open reading frames in the genome of Mtb have been classified as hypothetical proteins (Mazandu and Mulder, 2012). Determining the biological functions of these hypothetical proteins would undoubtedly improve comprehension of the Mtb life cycle. The hypothetical lipases/esterases such as Rv2030c, Rv1367c, Rv1922, Rv1063c and Rv3728, summarized in Table 2 and Supplementary Table S2, are predicted to be important in the life cycle in Mtb according to their subcellular localization and similarity to the lipolytic enzymes that have important roles in the Mtb.

**Table 2:** Hypothetical lipolytic enzymes of *M. tuberculosis* H37Rv and their predicted function.

Enzyme classification	Gene product	Predicted subcellular localization	Predicted function	Comments	References
Lipase	Rv1922	Extracellular	May contribute to loss of virulence	Similar to Mtb hypothetical proteins Rv1497(LipP), Rv2463(LipE), Rv3775(LipF), *M. bovis* Mb1957, etc. Contains PS00013 Prokaryotic membrane lipoprotein lipid attachment site	Dogra et al. (2015)
Esterase	Rv1062	Cytoplasmic	May contribute to loss of virulence	Similar to lipase and phospholipase	Ortega et al. (2016)
	Rv2565	Extracellular	Potential vaccine or drug targets Phosphatidylcholine metabolic process	Similar to *M. bovis* Mb2594	Knapp and Mcdonough (2014), Kumar et al. (2017c), and Johnson et al. (2020)
	Rv3728	Membrane protein	Involved in efflux system Lipid metabolic process Response to antibiotic Tetracycline transport; Cell wall and cell processes	Similar to *M. bovis* Mb3755	Knapp and Mcdonough (2014) and Kanji et al. (2018)
	Rv2030c	Extracellular	Response to antibiotic; Transferase activity; Nucleoside metabolic process; Predicted possible vaccine candidate	Similarity to *M. bovis* Mb2055c, Mb2056c	Mushtaq et al. (2015)
	Rv1367c	Extracellular	β-lactamase; Carboxylesterase; Possibly involved in cell wall biosynthesis	Some similarity to penicillin binding proteins, e.g., penicillin-binding protein 4 from *Bacillus subtili*; Similar to *M. bovis* Mb1402c	No information available

### 2.3 Phospholipases

The phospholipase A1 (PLA1), A2 (PLA2), C (PLC), and D (PLD) in the Mtb H37Rv phospholipase family hydrolyze phospholipids at various locations (Raynaud et al., 2002). Similar to the *Pseudomonas aeruginosa plc* genes, there are three adjacently positioned phospholipase C genes [*plc-a* (*rv2351c*)*, plc-b* (*rv2350c*)*, and plc-c* (*rv2349c*)] and a fourth truncated gene *plc-d* (*rv1755c*) that is situated elsewhere on the Mtb genome (Kong et al., 2005). The overall amino acid identity of PLC-A, PLC-B, and PLC-C is approximately 69%, while the amino acid identity of their C-terminal region is approximately 70% with PLC-D. The 227 amino acids in the N-terminal domain of PLC-D are absent in Mtb H37Rv. Furthermore, PLC-ABC exhibits between 30 and 40% amino acid sequence identity with PLC-H (hemolytic PLC) and PLC-N (nonhaemolytic PLC) from *Paeruginosa aeruginosa*. PLC-H and PLC-N have functions in the virulence of this pathogen *P. aeruginosa* (Ostroff et al., 1990; Guest et al., 2023).

PLC has been identified as a pathogenic component in many bacteria, including *Bacillus cereus* (Gilmore et al., 1989), *Clostridium perfringens* (Titball et al., 1989; Logan et al., 1991), *Listeria monocytogenes* (Boland et al., 1992) and *Pseudomonas aeruginosa* (Berka et al., 1981). All the recombinant PLC hydrolyze *p-*Nitrophenyl (NP) caproate: with the maximum specific activities (μmol min^−1^ mg^−1^) of 10.5 for PLC-A, 10.1 for PLC-B, 9.3 for PLC-C and 9 for PLC-D, respectively. Compared to PLCs of *B. cereus* and *Clostridium perfringens*, these specific activities of Mtb PLCs were 100 times lower (Johansen et al., 1996).

Parker et al. (2009) described mycobacterial phospholipase A activity (PLA), which was also a possible mycobacterial cutinase. The pathophysiology of disease and inflammatory states in humans are significantly influenced by PLAs, which hydrolyze phospholipids to fatty acids. PLA activity in Mtb has been demonstrated, and it has been discovered that this activity is connected to the cell wall and membrane fractions (Parker et al. 2009). These enzymes, which are most likely excreted from the Mtb cell wall, may cause the release of FA by hydrolyzing the phospholipids in the host cell membrane, supplying a carbon source and aiding in the activities involved in cell growth. It has been discovered that PLD activities occur in Mtb in addition to PLC and PLA activity. Although this enzyme may not be directly involved in virulence, because it is found in numerous species, including both pathogenic and non-pathogenic strains, it may nevertheless play a significant biological role in this genus of mycobacteria (Johansen et al., 1996; Gomez et al., 2001).

Rv0183 was identified as a lysophospholipase, which did not hydrolyze lysophospholipid substrates lysophosphatidylcholine but hydrolyzed monoacylglycerol substrates preferentially (Côtes et al., 2007). LipF (Rv3487c), a cell wall phospholipase C in Mtb hydrolyzes the phosphatidylcholine substrate.

### 2.4 Cutinases

Cutinases (EC 3.1.1.74), also known as cutinase-like proteins (CULPs) are serine esterases with Ser-His-Asp catalytic triad. Since they exhibit various characteristics shared by lipases and esterases, they are frequently identified as intermediaries between the two enzymes. Cutinases lack a hydrophobic “cap” or secondary structure covering their active site, in contrast to lipases However, a certain degree of flexible “mini-cap” present in the active site of cutinases (Longhi et al., 1997; Martínez and Maicas, 2021). These characteristics may allow the active site to adapt to varied substrates, including phospholipids and TAG, as well as big substrates like cutin.

There are at least seven genes called *cut* (1 to 7) which encode for the cutinase family in the Mtb genome. Although mycobacteria’s CULP members lack cutinase activity, they may be crucial in controlling various pathogenic processes (West et al., 2009). It has been discovered that Cut7 (Rv1984c) hydrolyzes medium-chain carboxylic esters, monoacylglycerols, and preferential phospholipids (Cole et al., 1998), whereas Cut4 (Rv3452) acts like phospholipase A2 (Schué et al., 2010).

## 3 The function of lipolytic enzymes in *Mycobacterium tuberculosis*

**Figure 1:**
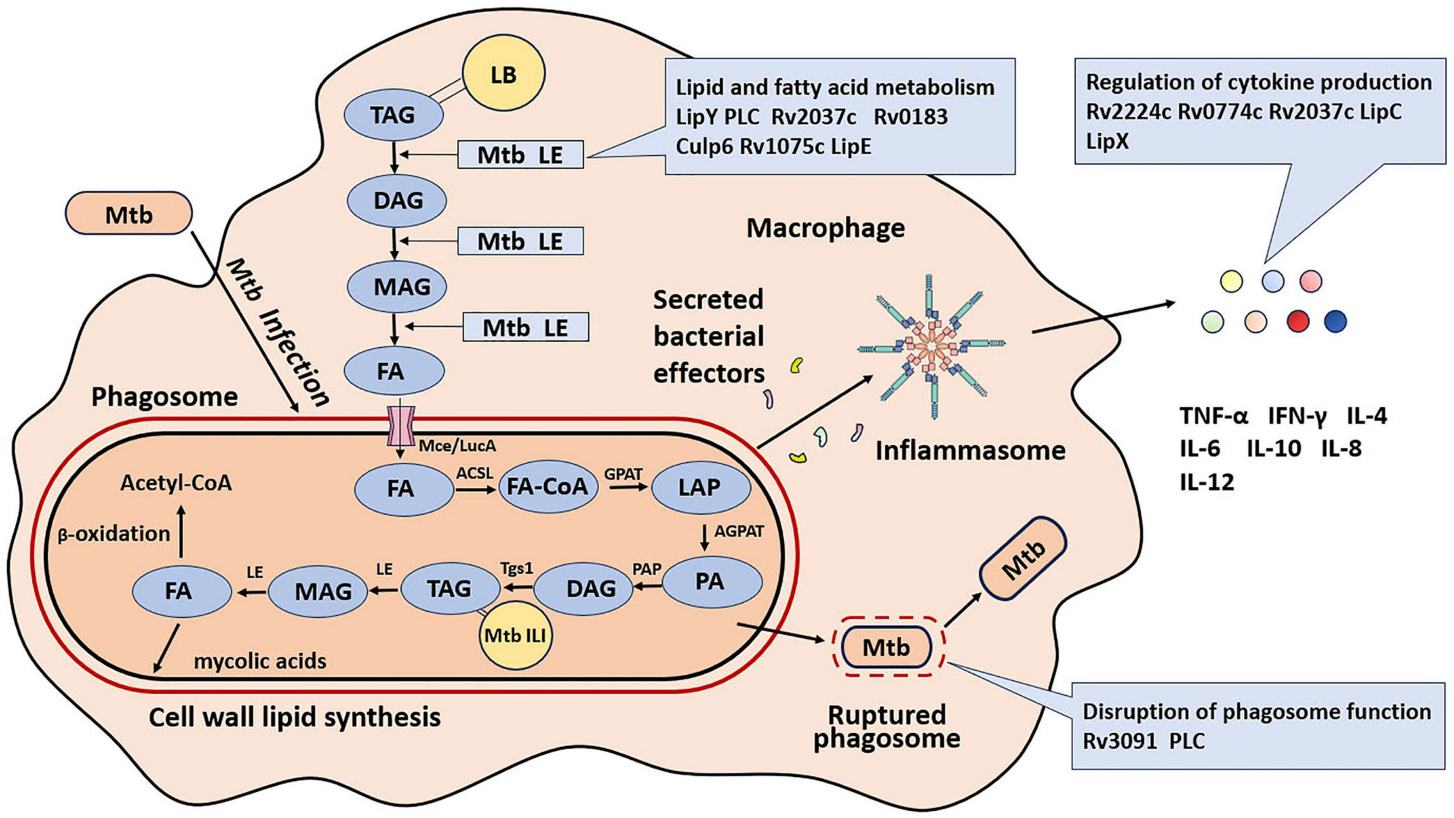
Roles of lipolytic enzymes in the pathogenesis of *M. tuberculosis*. LB, lipid inclusion body; TAG, triglyceride; FA, free fatty acid; Tgs1, triacylglycerol synthase 1; ILI, intracellular lipid inclusion; LE, lipolytic enzyme; DAG, diacylglycerol; MAG, monoacylglycerol; FA-CoA, free fatty acid acyl-coenzyme; GPAT, glycerol phosphate acyltransferase; AGPAT, acylglycerol-phosphate acyltransferase; PAP, phosphatidic acid phosphatase; Mce, multiprotein complex termed; LucA, lipid uptake coordinator A. LPA, lyso-phosphatidic acid; PA, phosphatidic acid.

Mtb has devoted a sizable amount of proteins to improving its survival, which is not surprising, given that it is an obligate intracellular pathogen. The current review focuses on Mtb lipolytic enzymes which significantly lower pathogenicity or virulence levels upon inactivation. Lipolytic enzymes promote mycobacterial survival by mediating lipid and fatty acid metabolism, disrupting phagosomes function, and regulating cytokine production (Figure 1).

### 3.1 Lipid and fatty acid metabolism

Global TB control efforts are significantly impacted by understanding the pathogen-host interaction in active tuberculosis. The role of exosomes in facilitating soluble mediator exchange and cell-to-cell interaction is increasingly acknowledged. These exosomes, released from the bacillus and infected host cells, contain lipids and proteins derived from both the host and Mtb. Lipidomics-based research has examined the lipid contents of exosomes from patients with active TB and healthy controls. The findings revealed the presence of triacylglycerols (TAG), free fatty acids, cholesterylesters (CE), phosphatidylcholines, phosphatidylinositols, and sphingomyelins (Sun et al., 2021; Biadglegne et al., 2022). TLC separation of extracted lipids from caseous granulomas, compared to healthy tissue, showed increased levels of CE, TAG, and cholesterol in lung biopsies from TB patients (Kim et al., 2010). A granuloma, formed when macrophages, lymphocytes, and dendritic cells aggregate at the infection site, is a major histopathological feature of TB. Macrophages within these granulomatous structures, in both experimental animal models and human disease, are termed foamy macrophages (FMs) (Ridley and Ridley, 1987; Cardona et al., 2000). It is demonstrated that FMs, laden with lipid bodies predominantly composed of TAG and CE, are crucial to the pathophysiology of TB (Peyron et al., 2008).

The extracellular lipolytic enzymes (LEs) of Mtb hydrolyze host lipids into fatty acids (FAs). These extracellular LEs break down extracellular host TAG into monoacylglycerol (MAG) and diacylglycerol (DAG) at varied rates, resulting in the release of free FAs (Côtes et al., 2008). FAs are imported into Mtb through a multiprotein complex known as the mammalian cell entry (Mce) system and lipid uptake coordinator A (LucA) (Nazarova et al., 2017; Wilburn et al., 2018). These free FAs are utilized by five enzymes, located at key points in the TAG metabolic pathway as depicted in Figure 1, to enhance the *de novo* synthesis of Mtb TAG as intracellular lipid inclusions (ILI). These enzymes include fatty acyl-coenzyme A (FA-CoA), glycerol phosphate acyltransferase (GPAT), acylglycerol-phosphate acyltransferase (AGPAT), phosphatidic acid phosphatase (PAP), and Triacylglycerol synthase 1 (Tgs1) (Low et al., 2010; Daniel et al., 2011). The accumulated Mtb TAG can be hydrolyzed by mycobacterial intracellular lipolytic enzymes during the phases of intracellular persistence and reactivation. The lipolytic enzyme LipY is capable of releasing fats from the accumulated TAG of Mtb for use during starvation (Deb et al., 2006). These fatty acids can then enter the β-oxidation pathway for energy production and be synthesized into mycolic acids, the major components of the Mtb cell envelope (Boshoff and Barry, 2005; Kremer et al., 2005; Dhouib et al., 2010; Singh et al., 2010; Hsieh et al., 2012; Lee et al., 2013). Overall, lipolytic enzymes are crucial for maintaining the pathophysiology of the bacteria, as illustrated in Figure 1.

LipY is capable of hydrolyzing ILI-containing TAGs (Deb et al., 2006; Mishra et al., 2008). Under nutrient-deficient conditions in Mtb, LipY expression is strongly induced, allowing the efficient utilization of accumulated TAGs without requiring an additional carbon source (Daniel et al., 2011). Among other members of the “Lip family,” such as LipC, LipL, LipK, and LipX, LipY demonstrates the highest specificity in degrading long-chain TAGs (Deb et al., 2006). LipY, following its solubilization from inclusion bodies, was purified. It exhibits optimal activity in hydrolyzing triolein, with *K*_*m*_ of 7.57 mmol L^−1^ and *V*_*max*_ of 653.3 nmol mg^−1^ min^−1^. The N-terminal region of LipY shows sequence homology with the proline-glutamic acid (PE) family protein, featuring polymorphic GC-rich repetitive sequences (Mishra et al., 2008). The C-terminal region of LipY is homologous to the HSL family and contains the conserved active-site motif GDSAG (Deb et al., 2006). LipY is a dual-cellular localization protein, found either intracytoplasmically or associated with the cell envelope. The PE N-terminal domain is cleaved by the ESX-5 secretion system. In the ΔLipY mutant, the capability to hydrolyze TAG was significantly reduced, highlighting this enzyme’s potential role in utilizing TAG during the dormancy and reactivation phases of Mtb (Deb et al., 2006). During active TB infection, the expression of LipY can be inferred from the detection of LipY-specific antibodies in patients (Daleke et al., 2011). LipY expression in macrophages reaches its peak within 24 hours following Mtb infection. In comparison with the wild-type H37Rv strain, mice infected with LipY-overexpressing H37Rv strain exhibited increased bacillary loads, exacerbated pathological conditions, weight loss, and higher mortality rates. Conversely, mice vaccinated with the recombinant LipY antigen demonstrated increased resistance to infection when challenged with the LipY-overexpressing strain. In these animal models, not only was there a decrease in Th1 and Th17 immune responses, but there was also an observed increase in the levels of regulatory T (T-reg) cells. These findings suggest that LipY plays a role in diminishing host defense mechanisms and augmenting the pathogenicity of Mtb (Singh et al., 2014).

Mtb possesses phospholipases C (PLC), which are critical for its pathogenicity in mice. PLC might perform several functions related to virulence, such as releasing fatty acids from host phospholipids. The upregulation of Mtb plc genes upon entry into phagocytic cells (Raynaud et al., 2002) and the comparatively high phospholipase activity in mycobacteria isolated from host tissues (Wheeler and Ratledge, 1991, 1992) align with this role. Several studies supporting this virulence-related function are as follows: Raynaud et al. (2002) found that triple and quadruple plc-knock-out mutants of Mtb were attenuated during later infection stages in mice, underscoring the role of PLC in mycobacterial persistence within the host. Furthermore, PLC-overexpressing Mtb strains showed increased survival compared to the PLC mutants (ΔPLC) when phosphatidylcholine was the sole nutrient source (Le Chevalier et al., 2015). Additionally, the hydrolytic action of the four Mtb PLCs on the host cell’s membrane phospholipids proved detrimental to mice macrophages (Bakala N’goma et al., 2010).

Phospholipase D (Rv2037c) utilizes lipids such as TAG, glycerol, and phosphatidylcholine from the host as an internal energy source to sustain infection and intracellular survival (Kumari et al., 2020). Rv2037c is a conserved transmembrane lipolytic enzyme characterized by a conserved pentapeptide (GXSXG motif). However, sequence alignment did not reveal the putative Ser-His-Asp triad typical of lipases; instead, only a Ser-Asp catalytic dyad was identified, a feature characteristic of PLD (da Mata Madeira et al., 2016). Cell wall modifications may be associated with enhanced resistance of the Rv2037c-overexpressing *M. smegmatis* strain to various stressors, including lysozyme, SDS, nutrient deprivation, acidic environments, and anti-TB medications (Kumari et al., 2020). The increased lipid content in the Rv2037c-overexpressing *M. smegmatis* strain, combined with damage to the outer membrane of macrophages and degradation of macrophage lipids by Rv2037c, collectively suggest its potential role in infection and intracellular survival.

Rv0183, localized at the cell wall, is involved in the hydrolysis of host cell lipids, as demonstrated by immunolocalization studies (Xu et al., 2010). It shares 36% and 34% amino acid sequence identity with rat and human monoglyceride lipases, respectively (Côtes et al., 2007). Homologues of Rv0183 have been identified in *M. leprae* (Ml2603, 79% sequence identity), *M. smegmatis* (MSMEG_0220, 68% sequence identity), and *M. bovis* (Mb0189, 100% sequence identity) (Rameshwaram et al., 2018). Rv0183, exhibiting a significant preference for monoacylglycerols, may act as a monoglyceride lipase, producing fatty acids for mycobacteria (Côtes et al., 2007). Using a disrupted mutant of the Rv0183 ortholog in *M. smegmatis*, MSMEG_0220, Dhouib et al. demonstrated Rv0183’s role in remodeling the mycobacterial cell wall. This mutant displayed a more homogenous culture with reduced cell clumping and a different colony morphology compared to the wild-type strain.

Culp6 (Rv3802c) is implicated in the production of mycolic acids, unique α-branched lipids present in the cell walls of mycobacteria. The bilayered cell wall of mycobacteria is integrated with these mycolic acids, which are essential for Mtb survival and play a significant role in cell signaling and evasion of host defenses, including granuloma formation (Parker et al., 2009). All mycobacterium genomes encode the cutinase family member Culp6, demonstrated to be vital for bacilli survival *in vitro* through transposon mutant construction (Sassetti et al., 2003). Parker et al. (2009) showed that Culp6, with thioesterase and phospholipase A (PLA) activity, hydrolyzes the phospholipid phosphatidylinositol mannoside (PIM). Since mycolic acid biosynthesis involves multiple ester and thioester bonds, these phospholipase and thioesterase activities align with its role in mycolic acid production. Furthermore, it has been observed that THL, an inhibitor of the human fatty acid synthase thioesterase (FASTE) domain similar to Rv3802c, reduces mycolic acid formation, leading to defects in the mycobacterial cell wall (Ravindran et al., 2014). However, the circumstantial evidence necessitates more definitive proof to confirm or refute Rv3802’s involvement in mycolic acid biosynthesis.

Rv1075c may play a role in lipid and fatty acid metabolism, providing carbon and energy when Mtb resides within host cells (Yang et al., 2019). Located at the cell wall and cell membrane of Mtb, Rv1075c is the only GDSL lipase reported in Mtb. It features a “GDSL” motif at the N-terminus, exhibits regiospecific activities, and demonstrates multifunctional substrate specificity (Akoh et al., 2004). The GDSL family is characterized by an active Ser-Asp/Glu-His site, with the active serine residue located at the N-terminus (Brick et al., 1995; Upton and Buckley, 1995). TesA of *Pseudomonas aeruginosa* PAO1, another GDSL-like lipase/acylhydrolase, shares 24.16% identity with Rv1075c (Kovacic et al., 2013). The transcriptional expression of rv1075c is enhanced at a lower pH (4.5), mimicking the acidic phagosome environment of macrophages. A rv1075c transposon insertion mutant strain showed reduced Mtb infection in mice, and bacterial growth in human peripheral blood mononuclear cell-derived macrophages and THP-1 cells was also dramatically decreased. Further research into Rv1075c’s role in lipid metabolism and Mtb’s intracellular survival could provide insights into how the bacterium utilizes host lipids/esters during *in vivo* infection and identify potential vulnerabilities for drug discovery.

LipE (Rv3775) is potentially involved in TAG metabolism in Mtb and crucial for intracellular survival. Yang et al. (2019) demonstrated that *lipE* expression is induced under stress conditions mimicking the intracellular environment of Mtb. Deb et al. (2006) found that a 12-day hypoxic growth of Mtb led to triglycerol accumulation, subsequently resulting in *lipE* upregulation. *LipE* can hydrolyze medium-chain triglycerol glyceryl trioctanoate. Additionally, Mtb ΔLipE showed a reduced bacterial burden in THP-1 cells, macrophages derived from human peripheral blood mononuclear cells, and mice infected with Mtb.

### 3.2 Disruption of phagosome function

Degradation of invasive Mtb by macrophage phagosomes is a crucial defense mechanism against Mtb infection. By interfering with the normal process of phagosome maturation, inhibiting acidification, and preventing their fusion with lysosomes, Mtb primarily infects macrophages and creates a replicative niche within these cells (Koul et al., 2004; Härtlova et al., 2018). The role of lipolytic enzymes in disruption of phagosome function is degrading the phagosomal membrane and modifying its permeability.

Several Mtb proteins are involved in disrupting proper functioning of the phagosome. Recently, Rv3091 was shown to permit an avirulent bacterium (*M. smegmatis*) to escape from a phagosome (Cui et al., 2020). The extracellular PLA Rv3091, which belongs to the patatin-like family in mycobacterium, displays the distinctive hydrolase α/β fold. Patatin was first discovered in potato tubers (Vancanneyt et al., 1989). The patatin domain includes an active site with a Ser-Asp catalytic dyad and an oxyanion hole stabilizing the enzyme-substrate transition state (Rydel et al., 2003). The nucleophilic serine in the patatin domain is situated in a tight turn between an α-heli and a β-sheet in a well-conserved β-β-α-β core structure, which it shares with mammalian lipases in a conserved core module (Schneider et al., 2006). Overexpressing *rv3091* in the surrogate *M. smegmatis* improves its capacity to survive, and the extracellular activity of Rv3091 promoted Mtb escape from the phagosome from macrophage phagosomes. *In vivo* experiments on mice demonstrated that the Rv3091 is involved in the pathogenicity of mycobacterium. The bacterial burden and damage to the lungs of infected mice were dramatically increased by the recombinant *M. smegmatis* strain that overexpressed the *rv3091*. Therefore, The PLA activity of Rv3091 enhances mycobacteria’s intracellular survival in macrophages, in addition to conferring phagosomal resistance. This protein also assisted the bacteria to use different lipids as the carbon source for their growth. Thus, Rv3091 protein could act as a potential target for the development of novel TB treatments (Cui et al., 2020).

Phospholipases C may also play the role of disrupting phagosome function by altering the permeability and degradation of the phagosomal membrane. However, Cavalier et al. (2020) showed that Mtb PLCs had no effect on virulence in the macrophages and mouse infection model, and that PLCs were not required for phagosomal rupture. The explanation is because mycobacterial phospholipases PLC are not released into the culture media, in contrast to other pathogenic bacteria (Marquis et al., 1995). Instead, they stay attached to the cell membrane. The location of these enzymes seems to contradict with their function in degradating phagosomal membrane. According to Wheeler and Ratledge (1992), this arrangement suggests that mycobacterial phospholipases have a non-aggressive role. This could eventually lead to the controlled release of fatty acids from the host, enabling intracellular mycobacteria to get nutrients without seriously harming the host. Chronic disease-causing mycobacterial agents might benefit from this characteristic.

### 3.3 Regulation of cytokine production

Mtb can fine-tune the innate immune response of the host to increase its virulence by generating inflammatory cytokines (Domingo-Gonzalez et al., 2016) (Figure 1). Table 3 summerizes some cytokines related to lipolytic enzymes, their receptors and respective roles in Mtb.

**Table 3:** The role of cytokines in *M. tuberculosis.*

Cytokine	Receptor/signal	Roles	References
TNF-α	TNFR1, TNFR2 JNK, p38, NFκB	Positive: Essential for survival following Mtb infection. Initiation of innate cytokine and chemokine response and phagocyte activation. Negative: Mediator of tissue damage.	Wajant et al. (2003) and Domingo-Gonzalez et al. (2016)
IFN-γ	IFNGR1, IFNGR2 JAK/STAT	Positive: Essential for survival following Mtb infection. Expressed by antigen-speciic T cells. Coordinates and maintains mononuclear inflammation. Negative: Potentially pathogenic.	Schroder et al. (2004) and Domingo-Gonzalez et al. (2016)
IL-4	IL-4R	Positive: Enhance B cell proliferation, differentiation and isotransformation. Negative: Inhibit macrophage and IFN-γ function	Nelms et al. (1999)
IL-6	IL-6R, gp130 JAK, STAT3, MAPK	Positive: Potentiates early immunity-nonessential unless a high-dose infection.	Heinrich et al. (2003) and Domingo-Gonzalez et al. (2016)
IL-10	IL-10R, TLR, MyD88	Negative: Inhibit the activation of macrophages, neutrophil B cells, mast cells, eosinophils, the production of Th1 cytokine.	Redford et al. (2011)
IL-8	CXCR1 CXCR2	Positive: Expressed on neutrophils mediates accumulation.	Yoshimura, 2015 and Domingo-Gonzalez et al. (2016)
IL-12	12Rβ1, IL-12Rβ2 JAK2, TYK2, STAT4	Positive: Essential for survival following Mtb infection. Mediate early T-cell activation, polarization, and survival. Negative: Overexpression of IL-12 is toxic during Mtb infection.	Vignali and Kuchroo (2012) and Domingo-Gonzalez et al. (2016)

Rv2224c (Hip1, CaeA) is a serine hydrolase located at the cell surface that prevents dendritic cells from producing a number of pro-inflammatory cytokines such as IL-12, IL-6 and TNF-α (Madan-Lala et al., 2014). Rv2224c has the catalytic triad S228-D463-H490 that is found in esterases, lipases, and proteases (Ferre and Clote, 2005). Rv2224c, as an esterase/lipase, is preferentially hydrolyzing ester bonds of substrates with about 3 to 7 carbon atoms chain length (Lun and Bishai, 2007). It has been identified as a critical immunomodulatory protein that inhibits robust macrophage activation after Mtb infection. It regulates the initiation and intensity of pro-inflammatory responses (Rengarajan et al., 2008; Madan-Lala et al., 2011; Naffin-Olivos et al., 2014). The pathogen is expected to benefit from suppressing early pro-inflammatory responses because it will be able to evade immune identification (Vandal et al., 2009). Rv2224c and its orthologue from *M. smegmatis* are crucial for preserving the integrity of the cell envelope and conferring resilience to stressors. Furthermore, the GroEL2 protein, which is an immunomodulatory protein, is a substrate of Rv2224c (Naffin-Olivos et al., 2014). GroEL2 encodes a chaperone-like protein and is cleavaged to a monomeric form from a multimeric form. Even though GroEL2 remains uncleaved in the Rv2224c mutant strain, ectopic synthesis of cleaved GroEL2 monomers in this strain restores wild type levels of cytokine responses in infected macrophages. It is suggested that Rv2224c-dependent proteolysis substrate is a unique regulatory mechanism in Mtb as it enables the pathogen to quickly adapt to shifting immunological settings in the host during infection (Naffin-Olivos et al., 2014). The role of Rv2224c in the virulence of Mtb due to its role as esterase/lipase are needed to further study.

Rv0774c may contribute to mycobacterium’s ability to avoid the extremely harsh environment in the macrophages by inhibiting host’s protective response and remodeling the cell wall lipid. The expression of Rv0774c in *M. smegmatis* led to substantial upregulation of the TLR2 receptor (Toll-Like Receptor) and IL-10 cytokine production. However, the production of pro-inflammatory cytokines such IL-12, TNF-α, IFN-γ and MCP-1 were reduced (Kumar et al., 2017b). Rv0774c may be involved in the surface mycolation of trehalose monomycolate to create trehalose dimycolate via mycolylmannosylphosphorylheptaprenol. The function of Rv0774c involved in this cell wall lipid remodeling conferred altered morphology and increased streptomycin resistance. Overall, Rv0774c expression alters the survival of *M. smegmatis* in macrophages while also changing the associated immunological response (Kumar et al., 2017b). An effective therapeutic target for the treatment of tuberculosis may be the heightened anti-inflammatory response, which could be one of the causes of bacterial persistence inside macrophages. However, the interactions between various pathways in Mtb and *M. smegmatis* are different. Therefore, gene knock out and animal studies could be used to confirm the importance of Rv0774c in enhancing the virulence of Mtb in the future.

Many lipolytic enzymes have other functions related to the pathogenicity and virulence of Mtb in addition to those mentioned above. Phospholipase Rv2037c causes BALB/c mice, a kind of immunodeficient mouse widely used in the study of oncology, physiology, immunology, to produce pro-inflammatory cytokines such IL-8, IL-12 and TNF-α, thereby suggesting its role in immune-modulation. Rv0183 dramatically increased apoptosis and inflammatory markers like IL-6, NF-B, TLR2, TLR6, TNF-γ and MyD88 when it was ectopically expressed in murine macrophages (Xu et al., 2010). LipC (Rv0220), a cell surface esterase, has immunogenicity and can induce the production of proinflammatory cytokines and chemokines such as IL-8, IL-12, TNF-α and MCP-1 in macrophages and lung epithelial cells (Shen et al., 2012).

LipX regulates the secretion of macrophage IL-6 and ultimately contributes to the cell death of the macrophage (Deng et al., 2015; Singh et al., 2016; Rastogi et al., 2017). LipX (Rv1169c) is a member of the PE family, which is specific to pathogenic mycobacteria (including Mtb and *M. bovis*) but absent in non-pathogenic *M. smegmatis*. LipX is located in the cell wall of Mtb and is surface-exposed, which may play a role in the hydrolysis of host lipids (Cascioferro et al., 2007). Anti-LipX antibodies are observed in TB patients (Narayana et al., 2007) and human lung granulomas (Sampson, 2011). This suggests that LipX may be overexpressed during an active Mtb infection. Additionally, the expression levels of LipX increased under conditions of acidic stress, adaptation to stationary phase, starvation, and in hypoxic lipid-loaded macrophages (Schnappinger et al., 2003; Voskuil et al., 2004; Daniel et al., 2011). Mtb ΔLipX exhibited lower survival in activated THP-1 macrophages compared to the wild type Mtb strain (Rastogi et al., 2017). According to all of these investigations, LipX is crucial for mycobacterial pathogenicity.

## 4 Lipolytic enzymes as biomarkers, drug targets, and vaccine candidates

The rapid emergence of antibiotic resistance in bacteria calls for the development of new, powerful antibiotics for treating infections. Lipolytic enzymes are now emerging as potential targets for new generation of treatments. This is due to their crucial roles in virulence and survival, particularly in the case of Mtb (Kim and Shin, 2023). Lipolytic enzymes play a significant role in hydrolyzing host lipids into fatty acids that provides energy to the bacilli, serves as a major nutrition source during dormancy and reactivation phases, as well as serves as the only source of precursors for the synthesis of the cell wall. In addition to their potential as therapeutic agents, lipolytic enzymes of Mtb could also serve as biomarkers in the serodiagnosis of active tuberculosis (Brust et al., 2011).

According to a research by Low et al., TAGs are extensively accumulated and degraded in bacilli as they enter and exit hypoxia-induced dormancy, respectively (Low et al., 2009). Additionally, these actions are accompanied by the dynamic emergence and disappearance of TAG lipid particles inside cells (Dhouib et al., 2011). Regrowing bacilli exhibit a notable correlation between reduced TAG levels and elevated cellular TAG lipase activity, suggesting that TAG usage plays a crucial role in mycobacteria’s ability to proliferate again after emerging from the non-replicating stage (Low et al., 2009). As a result, lipolytic enzymes like LipY, which are primarily generated and produced during reactivation conditions and are not expressed under normal growth settings, may serve as helpful biomarkers to identify reactivated forms of tuberculosis (Mishra et al., 2008). Lipolytic enzymes Rv0183, Rv1984c and Rv3452 cause TB patients to have strong humoral reactions. In particular, Rv3452 showed excellent serodiagnostic qualities in both populations, indicating that this marker will be highly valuable for diagnostic purposes in the future.

Inhibitors that target lipolytic enzymes appear to be potential therapeutics against Mtb. Tetrahydrolipstatin is a pancreatic lipase inhibitor pharmacophore that was used to develop chemical inhibitors against cell wall lipase Rv3802c. These inhibitors exhibited antibacterial activity *in vitro*, indicating that Rv3802c may be a promising therapeutic target for Mtb (West et al., 2011). Kumari et al. (2020) indicated that Rv2037c is a phospholipase that can be potentially used for the creation of innovative drugs for TB treatment (Kumari et al., 2020). Rv0183 is a monoacylglycerol lipase (MGL) that has potential as a druggable target. Thiadiazole carbamate compound lalistat is a particular inhibitor of human lysosomal acid lipase. It has been shown to hinder the *in vitro* development of Mtb. Lipolytic enzymes such as LipI, G, M, N, and O were found to be the targets of lalistat (Rameshwaram et al., 2018). LipX is speculated to act on the ultimate hydrolyzed product of TAG to release free fatty acids that serve as the building blocks for maintaining and modifying the cell wall of Mtb in a hostile environment (Singh et al., 2016). LipX has been determined as essential for mycobacterial pathogenicity and it enhances resistance to various environmental stresses that bacteria experience in the phagosome (Singh et al., 2016). Therefore, LipX function-inhibiting small molecules may be clinically valuable in controlling Mtb infections.

Culp1(Rv1984c), Culp2 (Rv2301) and Culp6 (Rv3802c) are thought to be novel targets for the TB vaccine since they induce IFN-γproduction (Shanahan et al., 2010). Compared to Culp1and Culp6 alone, Culp1-6 fusion protein showed an higher level of protection against infection (Shanahan et al., 2010). The localization of Rv3097c has the potential for creating recombinant mycobacteria expressing heterologous antigens on their surface in order to product vaccine (Cascioferro et al., 2007). Other surface lipolytic enzymes mentioned in this review have the potential as vaccines.

## 5 Discussion

One of the main factors contributing to Mtb pathogenesis is capability of this bacteria to elude immune destruction and endure in macrophages, where it eventually results in chronic infection. However, the pathogenesis mechanism of Mtb is still unknown, making TB diagnosis and treatment difficult. Understanding the molecular mechanism of pathogenicity, virulence, and persistence has advanced significantly in recent years. The discovery of crucial proteins responsible for mycobacterial pathogenicity has been one important contribution. The majority of these virulence proteins are involved in lipid metabolism and signal transduction pathways (Forrellad et al., 2013).

Mtb depends on lipids for growth and virulence expression. Foamy macrophages gather lipids in granulomas during Mtb infection, giving Mtb metabolic adaption and survival strategies against various challenges. Antibiotics that target the bacterial cell wall or transcription may become less effective against drug-tolerant Mtb due to the involvement of host-derived lipid molecules, such as cholesterol and triacylglycerol. The metabolism of lipids is significantly influenced by lipolytic enzymes.

In this review, We have classified lipolytic enzymes in to four kinds: lipases, esterases,cutinases and phospholipases, but still there are other kinds, such as β-lactamases enzymes, PE/PEE family and HSL family. β-lactamases (EC 3.5.2.6), with the consensus sequence S-X(T)-X(S)-K, are responsible for their resistance to β-lactam antibiotics such as cephalosporin, penicillin, carbapenem(ertapenem) and cephamycin. A four-atom ring known as a beta-lactam ring unites the molecular structures of these antibiotics. The ring is broken by the lactamase enzyme, rendering antibacterial effects of β-lactam antibiotics inactive (Hugonnet et al., 2009). LipD, LipE, LipL, and LipP exhibit β-lactamases activity (Supplementary Table S1).

The existence of two multigene families that combined account for about 10% of the chromosomal coding potential is one of the Mtb genome’s most intriguing features. The conserved N-terminal regions of these two protein families, known as PE and PPE, are approximately 100 and 180 amino acids long, respectively (Cole et al., 1998). While PPE stands for the Pro-Pro-Glu motif, which is often found in the first 10 amino acids of these proteins, PE is named after the characteristic motif Pro-Glu. Of these two families, PE is the biggest. PE-PGRS with a C-terminal PGRS (Polymorphic GC-rich Repetitive Sequences) domain is the the largest subfamily of PE. LipX, also known as PE11, belongs to the PE family. LipY belongs to the PE-PGRS family. It has been shown that PE and PPE increase bacterial survival and alter human immunity, cell death, and metabolism (Yan et al., 2020).

Lipolytic enzymes are extremely flexible weapons that mycobacteria can use. There is growing evidence for their functions in (i) lipid and fatty acid metabolism, (ii) disruption of phagosome function, and (iii) regulation of cytokine production. Actually, we also can discover that many lipolytic enzymes have more than one roles in the virulence and pathogenicity of Mtb. PLC performs a number of virulence-related roles. First, PLC may release fatty acids from host phospholipids, which could supply the bacteria with nutrients. Second, PLC has the potential to completely destroy the phagosomal membrane or alter its permeability. Lastly, PLC may alter the host immune responses by interfering with signal transduction processes in infected cells through the activation of the arachidonic acid cascade. In addition to lipid metabolism, many lipolytic enzymes can induce immune responses from the host but the detailed mechanism still needs to be explored.

This review has taken into account bioinformatics prediction data, however it primarily focuses on lipolytic enzymes whose particular roles in virulence have been established. There are still a large number of hypothesized lipolytic enzyme genes that need to be studied for physiological properties and functions (Supplementary Table S2). There are other functions of this enzymes that we have not introduce certainly because of the less resports, such as LipX, It has been reported to induce necrosis in the host with unknown mechanism (Deng et al., 2015; Rastogi et al., 2017).

In conclusion, Despite lipolytic enzymes have important roles in in the virulence and pathogenicity of Mtb, there are only a limited number of accessible in-depth studies. Current research has largely focused on a small number of well-established lipolytic enzyme family members, such as Lip family. Through more research in the future, a deeper understanding of the functions of these fascinating mycobacterial lipolytic enzymes might be achieved. More studies on how they relate to Mtb-host interaction, Mtb survival, and Mtb pathogenesis should also be undertaken. Given the reported roles of certain well-known lipolytic enzymes so far, we predict the discovery of more effector lipolytic enzymes in the future.

## Author contributions

HL: Writing – original draft. JX: Writing – review & editing. HW: Writing – review & editing. SW: Writing – review & editing. RF: Writing – review & editing. XL: Writing – review & editing. ZL: Writing – review & editing. NS: Writing – review & editing.
