# Supplementary material for: Roles of Lipolytic enzymes in Mycobacterium tuberculosis pathogenesis
Source: Front Microbiol. 2024 Jan 29;15:1329715. doi: 10.3389/fmicb.2024.1329715 (PMC10865251; doi:10.3389/fmicb.2024.1329715)
Supplement: Supplementary file 1 [file Table_1.docx]

Supplementary Material

# Supplementary Tables

**Supplementary Table S1. Lipolytic enzymes of *M. tuberculosis* H37Rv and their function**

| **Enzyme classification** | **Gene Product** | **Subcellular localization** | **Enzymatic activity** | **Function** | **References** |
| --- | --- | --- | --- | --- | --- |
| Lip family | LipC (Rv0220) | **Cell wall and capsule | **Esterase  **Hydrolysis of short-chain *p-*NP esters (C_2_-C_10_)  Specific activity :  160 mU mg^−1^  *K_m_* : 2.89 mmol L^-1^  *V_max_* : 0.48 μmol L^-1^ min^−1^ | **Macrophages and pulmonary epithelial cells are stimulated by LipC to release pro-inflammatory cytokines and chemokines | Shen et al. (2012) |
|  | LipD (Rv1923) | *Periplasmic | **Esterases, lipase, β-lactamase  **Hydrolysis of *p-*NP palmitate  Specific activity :  16 U mg^−1^  *K_m_* : 0.645 mmol L^-1^  *V_max_* : 24.75 U mL^−1^  *k_cat_* : 0.02112 s^-1^ | **LipD is upregulated in oxidative stress conditions  **LipD is active and stable at high temperatures and low pH | Singh et al. (2010, 2014) |
|  | LipE (Rv3775) | *Periplasmic | **Carboxylesterase  **With the maximal activity on hexanoate  **β-Lactamase  Specific activity :  560.95 U mg^−1^  *Km* : 711.58 mol L^-1^  *V_max_* : 6756.76  mmol L^-1^ min^−1^  *K_cat_* : 7.7685 s^-1^ | ***In vivo* infection and intracellular growth are both facilitated by LipE | Singh et al. (2010), and Yang et al. (2019) |
|  | LipF (Rv3487c) | *Cell wall | **Carboxylesterase  **The optimal activityfor triacetin and *p-*NP acetate  **Phospholipase C activity  Specific activity :  176 mU mg^−1^ | **LipF is expressed at low pH  **Related to virulence of this pathogen | Zhang et al. (2005), Delorme et al. (2012) |
|  | LipG (Rv0646c) | **Periplasmic | **Phospholipase and thioesterase activities  **Esterase  Specific activity:  22 U mg^−1^  *K_m_* : 500 µmol L^-1^  *V_max_* :  58.82 µmol min^−1^ mL^−1^  Catalytic efficiency : 3.92 µmol L^-1^ min^−1^ | *LipG has a probable function in lipid metabolism  **LipG up-regulated the production of various pro-inflammatory cytokines (TNF-α and IFN-γ), chemokine (IL-8), and nitric oxide in THP-1-derived macrophages  **LipG is involved in modification and remodeling of the mycobacterial envelope | Singh et al. (2010), Rastogi et al. (2018), and Cavalier et al. (2020 ) |
| * Bioinformatics prediction.  **Experimentally defined. | | | | | |

| **Supplementary Table S1.** **Lipolytic enzymes of *M. tuberculosis* H37Rv and their function (cont.)** | | | | | |
| --- | --- | --- | --- | --- | --- |
| **Enzyme classification** | **Gene Product** | **Subcellular localization** | **Enzymatic activity** | **Function** | **References** |
| Lip family | LipH (Rv1399c) | *Cytoplasmic | **Carboxylesterase  Specific activity : 1350 U mg^−1^ | *LipH has a probable function in lipid metabolism | Canaan et al. (2004), Singh et al. (2010), and Delorme et al. (2012) |
|  | LipI (Rv1400c) | *Cytoplasmic | **Carboxylesterase  **Hydrolysis of  short-chain *p-*NP and vinyl butyrate  Specific activity : 73 U mg^−1^ | *LipI likely has a role in lipid metabolism | Delorme et al. (2012), and Lin et al. (2017) |
|  | LipJ (Rv1900c) | **Cytoplasmic and extracellular | **Esterases  Specific activity :  397 U mg^−1^ | **A connection between Rv1900c and stress tolerance through cell wall modulation,  **The expression of *rv1900c* was upregulated under acidic, nutritive and iron stress conditions in Mtb | Kumari and Kaur (2021 ) |
|  | LipK,  MBTJ  (Rv2385) | *Cytoplasmic | **Esterase/acetyl  hydrolase | **Its expression was upregulated under iron stress  **Expression of *mbtJ* changed colony morphology and enhanced the growth/infection in *M. smegmatis* | Singh et al. (2010), and Chownk et al. (2018 ) |
|  | LipL (Rv1497) | *Cytoplasmic | **β-lactamase  **Esterase  **Hydrolysis of pNP butyrate  *K_cat_* : 0.0932 s^-1^  Specific activity : 0.32 U mg^−1^ | **LipL is elevated in situations of acidic, oxidative, and nutritional stress, pointing to its physiological function in the bacilli life cycle | Cao et al. (2015), Singh et al. (2016), and Dey et al. (2022) |
|  | LipM (Rv2284) | *Cytoplasmic | *Probable esterase | *LipM is likely involved in lipid hydrolysis and metabolism  **lipM were also induced during nutrient starvation  **LipM remained active in dormant Mtb | Singh et al. (2010), Tallman et al. (2016), and Arya et al. (2022 ) |
|  | LipN (Rv2970c) | **Cytoplasmic | **Carboxylesterase  **Hydrolysis of pNP butyrate  **The highest catalytic efficiency against small carbon esters, including methyl ether, ethyl ether, and oxazole esters  Specific activity : 217 U mg^−1^ | **LipN is expressed in macrophages under acidic stress both *in vitro* and *in vivo* after 6 h of infection  **LipN remained active in dormant Mtb | Delorme et al. (2012), Jadeja et al. (2016), Tallman et al. (2016), and Johnson et al. (2020) |
|  | LipO (Rv1426c) | *Cytoplasmic | *Possible esterase  *Possible deacetylase | *LipO is likely involved in lipid metabolism | Singh et al. (2010) |
|  | LipP (Rv2463) | *Periplasmic | **β-Lactamase family  *Probable esterase | **Expression in macrophages at the earliest stages of infection that led to suppressive reactions in both dendritic cells and macrophages | Singh et al. (2010) |
| * Bioinformatics prediction.  **Experimentally defined. | | | | | |

| **Supplementary Table S1.** **Lipolytic enzymes of *M. tuberculosis* H37Rv and their function (cont.)** | | | | | |
| --- | --- | --- | --- | --- | --- |
| **Enzyme classification** | **Gene Product** | **Subcellular**  **localization** | **Enzymatic activity** | **Function** | **References** |
| Lip family | LipQ (Rv2485c) | *Cytoplasmic | **Lipase | **When compared to LPS-stimulated macrophages, rLipQ dramatically increases the amount of anti-inflammatory cytokines like IL-4 and IL-10 while significantly decreasing the level of pro-inflammatory cytokines (TNF-α and IFN-γ)  **Prevent LPS-stimulated macrophages from expressing iNOS, TLR-2, and the transcription factor NF-kB  **Only under conditions of oxidative stress was expressed | Singh et al. (2010) Kumar et al. (2017) |
|  | LipR (Rv3084) | *Cytoplasmic | **Alkaline eurythermic esterase  **Optimal substrate *p-*NP acetate  Specific activity :  425 mU mg^-1^  with *p-*NPC8 | **LipR is increased under low pH, suggesting that it plays a role in mycobacteria's ability to block phagosomal acidification or in bacilli's ability to survive intracellularly  **LipR could inhibit the secretion of interferon-γ (IFN-γ) and interleukin-2 (IL-2), but to stimulate the secretion of IL-10 | Sheline et al. (2009) Singh et al. (2010), Delorme et al. (2012), Fisher et al. (2002), and Zhang et al. (2019) |
|  | LipS  (MesT,  Rv3176c) | **Membrane | **Esterases/epoxide hydrolase  **Highly specific for (R)-ibuprofen-phenyl ester  Specific activity :  200 U mg^-1^ with  *p-*NP acetate | **LipS was upregulated under hypoxic conditions  *Virulence, detoxification, and adaptability are likely involved | Chownk et al. (2017) |
|  | LipT (Rv2045c) | *Cytoplasmic | **Triacylglycerol lipases | *LipT is likely involved in lipid metabolism | Singh et al. (2010) |
|  | LipU (Rv1076) | **Extracellular | ***p-*NP butyrate as preferred substrate  Specific activity :  177 U mg^−1^  *K_m_*: 334 μmol L ^-1^  *V_max_*:  262 μmol mL^−1^ min^−1^ | **The upregulation of Mtb *rv1076*  under nutritive stress implicates a role in starvation.  **Rv1076 elicited strong humoral response in both extrapulmonary and relapsed cases of TB patients | Grifﬁn et al. (2011), Delorme et al. (2012), Li et al. (2017), and kaur et al. (2017) |
|  | LipV (Rv3203) | **cell wall | **Esterase/lipase  **Hydrolysis of *p-*NP myristate  Specific activity : 21.29 U mg^−1^  *K_m_* : 714.28 μmol L^-1^  *V_max_* : 62.5 μmol ml^−1^ min^−1^ | **LipV is upregulated under conditions of acidic stress, demonstrating its significance in Mtb survival in an acidic environment  **An upregulation of cytokines with a Th-1 bias, and enhanced expression of co-stimulatory markers on both antigen-presenting cells and T lymphocytes | Singh et al. (2014), and Mohammad et al. (2016) |
| * Bioinformatics prediction.  **Experimentally defined. | | | | | |

| **Supplementary Table S1.** **Lipolytic enzymes of *M. tuberculosis* H37Rv and their function (cont.)** | | | | | |
| --- | --- | --- | --- | --- | --- |
| **Enzyme classification** | **Gene Product** | **Subcellular**  **localization** | **Enzymatic activity** | **Function** | **References** |
| Lip family | LipW (Rv0217c) | *Cytoplasmic | **Esterase  **Hydrolysis of *p-*NP butyrate  Specific activity: 1600 mU mg^−1^ | *LipW is likely involved in cellular metabolism and alkaloid biosynthesis II | Singh et al. (2010), and Delorme et al. (2012) |
|  | LipZ (Rv1834) | *Cytoplasmic | *Probable hydrolase | *Respiration and intermediate metabolism are likely to be impacted by LipZ | Singh et al. (2010) |
| Cutinase | Culp1 /Cut7  (Rv1984c) | **Extracellular | **Esterase  ** Phospholipase  **Hydrolysis of *p-*NP butyrate and vinyl esters  Specific activity :  86 U mg^-1^ with  *p*-NP caprylate  *V_max_* : 1688  pmol min^−1^ mg^−1^ | **Cut7 stimulates IFN-γ and IL-12 cytokine production as well as T cell proliferation  **As a protein vaccine, Cut7 is highly immunogenic and protective | Grover et al. (2006), West et al. (2008, 2009), and Schué et al. (2010) |
|  | Culp2/Cut2  (Rv2301) | **Extracellular | **Esterase  **Hydrolysis of *p-*NP butyrate | **Cut2 stimulates T lymphocytes that secrete IFN-γ  **Highly protective and immunogenic as a protein vaccination | West et al. (2008, 2009; Ocampo et al., 2012) |
|  | Culp3/Cut3  (Rv3451) | **Extracellular | **Esterase and Lipase  **Hydrolysis of *p-*NP butyrate  **TDM hydrolase | **Through the hydrolysis of TDM, Cut3 alters the permeability of the cell membrane and boosts nutrient input  **Secrete IFN-γ | West et al., 2008, 2009), and Yang et al. (2014) |
|  | Culp4/Cut4  (Rv3452) | **Cell wall | **Esterase  **Phospholipase A2  **Hydrolysis of  phospholipids  Specific activity : 50×10^-3^U mg^-1^ with *p-* NP butyrate | **Cut4 participates in the modification of the Mtb cell membrane and the hydrolysis of TDM  **Cut4 causes lysis of macrophages | Schué et al. (2010), and Yang et al. (2014) |
|  | Culp5/Cut1  (Rv1758) | *Cytoplasmic | *Probable cutinase | –*Cut1 most likely contributes to the hydrolysis of cutin | West et al. (2009) |
|  | Culp7/Cut5b  (Rv3724B) | **Cell wall,  cytoplasmic,  membrane | *Probable cutinase | *Cutin hydrolysis is likely mediated by Culp7 | Verma et al. (2015) |
| Other  lipases/  esterases | Rv1592c | *Periplasmic | **Lipase  **Hydrolysis of *p-*NP laurate  Specific activity : 117.3 U mg^-1^ | **Its expression was up-regulated during isoniazid (INH) treatment | Sassetti et al. (2003), and Kumar et al. (2017, 2021) |
|  | Rv0774c | **Extracellular | **Esterase  **Hydrolysis of  *p-*NP octanoate  Specific activity :  7.1 U mg^-1^ | **Rv0774c induced by iron stress, plays a role in immune suppression linked to altered cytokine and TLR2 expression | Kumar et al. (2017) |
| * Bioinformatics prediction.  **Experimentally defined. | | | | | |

| **Supplementary Table S1.** **Lipolytic enzymes of *M. tuberculosis* H37Rv and their function (cont.)** | | | | | |
| --- | --- | --- | --- | --- | --- |
| **Enzyme classification** | **Gene Product** | **Subcellular**  **localization** | **Enzymatic activity** | **Functions** | **References** |
| Other  lipases/esterases | Rv1075c | **Cell wall and membrane | **GDSL-like esterase  **Hydrolysis of triacetin and tributyrin  With *p*-NP acetate  Specific activity : 2692.62 U mg^-1^  *K*_m_ : 753.59 μmol L^-1^  *V_max_* : 135.15  mmol L^-1^ min^-1^ | **Its expression was induced at pH(4.5), simulating the acidic phagosome of macrophages  **Rv1075c mutation decreased bacterial growth in THP-1 cells and attenuated Mtb infection in mice  **Inside host cells, Rv1075c contributes to the metabolism of esters and fatty acids | Yang et al. (2019) |
|  | Rv2037c | **Membrane and extracellular | **Lysophospholipase  **Lipase  Specific activity:  200 U mg^-1^  with  *p*-NP decanoate | ***rv2037c* gene expression was up-regulated under acidic and nutrient stress in Mtb H37Ra  **Rv2037c increased the survival of *M. smegmatis* *in vivo*/*ex vivo* and stimulated the production of pro-inflammatory cytokines | Kumari et al. (2020) |
|  | Rv1683 | **Cell membrane | **Lipase  **Acyl-CoA synthase | *Rv1683 is likely involved in the biosynthesis and breakdown of lipids  **Rv1683 is associated with D-cycloserine resistance | Low et al. (2010), and Chen et al. (2017) |
|  | Rv3036c | **Cell wall  and membrane | **Esterase  **Hydrolysis of *p-*NP acetate  Specific activity : 1315.97 U mg^-1^ | *Possible impact on cell wall and cellular functions | Chen et al. (2014) |
|  | Rv0045c | *Cytoplasmic | **Esterase  **Hydrolysis of *p-*NP caproate  Specific activity :  3.5 U mg^-1^ | *Rv0045c most likely contributes to lipid metabolism | Guo et al. (2010), and Bowles et al. (2021) |
|  | Rv1430  (PE16) | *Inner  membrane | **Esterase  **Hydrolysis of *p-*NP caproate  Specific activity : 22.5 U mg^-1^ | *Probably involved in lipid metabolism | Sultana et al. (2013) |
|  | Rv0519c | **Cell membrane | **Esterase/lipase  Specific activity :  17 U mg^-1^ with  *p*-NP butyrate | **Drug resistance | Srivastava et al. (2008), and Kumar et al. (2017) |
|  | Rv3539  (PPE63) | **Cell  membrane  and  extracellular | **Esterase activity.  with the *p*-NPC4 as optimum substrate | **Rv3539 improved intracellular survival of *M. smegmatis* via cell wall modulation and altered immune response of host | Anand and Kaur (2023), and Anand et al. (2023) |
|  | Rv3091 | **Extracellular | **Phospholipase A1, phospholipase A2, and lipase activity  Specific activity : 2000 U mg^-1^ with  *p*-NP butyrate | **It increases the intracellular survival of mycobacterium by enabling it to use phospholipids for growth and providing resistance to phagosome destruction | Cui et al. (2020) |
| * Bioinformatics prediction.  **Experimentally defined. | | | | | |
