# Supplementary material for: Roles of Lipolytic enzymes in Mycobacterium tuberculosis pathogenesis
Source: Front Microbiol. 2024 Jan 29;15:1329715. doi: 10.3389/fmicb.2024.1329715 (PMC10865251; doi:10.3389/fmicb.2024.1329715)
Supplement: Supplementary file 2 [file Table_2.docx]

Supplementary Material

# Supplementary Tables

**Supplementary Table S2.** **Hypothetical lipolytic enzymes of *M. tuberculosis* H37Rv and their predicted function**

| **Enzyme classification** | **Gene product** | **Predicted**  **subcellular**  **localization** | **Predicted function** | **Comments** | **References** |
| --- | --- | --- | --- | --- | --- |
| Esterase  /lipase | Rv1063c | Extracellular | No information available. | Partially similar to Rv3239c,  *M. bovis* Mb1092c,  *M. smegmatis* MSMEG_5284, etc | No information available |
|  | Rv3239c | Cell wall and cell processes | Seems involved in efflux system. | Similar to Rv2565, *M. bovis* Mb3267c, equivalent to Rv3728\| putative two-domain membrane protein | No information available |
|  | Rv0399c | membrane protein | β-lactamase family protein | Some similarity to penicillin binding proteins and various peptidases. Also similar to other Mtb PBPs and esterases | No information available |
|  | Rv0671 | membrane protein | PHB depolymerase family; Esterase;  depolymerase | Highly similar to Rv3298c putative lipoprotein from *M. tuberculosis*. Also similar to a large variety of proteins including various esterases and *M. bovis* Mb0405c | No information available |
|  | Rv1639c | Membrane protein | No information available | No information available | No information available |
|  | Rv1367c | Extracellular | β-lactamase;  Carboxylesterase;  possibly involved in cell wall biosynthesis | Some similarity to penicillin binding proteins e.g. penicillin-binding protein 4 from *Bacillus subtili*；Similar to *M. bovis* Mb1402c | No information available |
|  | Rv3593 | Cell wall | Structural and mechanistic studies on a cephalosporin esterase from the clavulanic acid biosynthesis pathway | Equivalent to AAK48057 from Mtb strain CDC1551 and *M. bovis* Mb3624 | Jeon et al. (2015) |
|  | Rv1835c | Extracellular | No information available | Similarity to putative acylases e.g. G216374 glutaryl 7-aca acylase precursor | Norheim et al. (2017) |

| **Supplementary Table S2.**  **Hypothetical lipolytic enzymes genes of *M. tuberculosis* H37Rv and their predicted function (cont.)** | | | | | |
| --- | --- | --- | --- | --- | --- |
| **Enzyme classification** | **Gene Product** | **Predicted**  **subcellular**  **localization** | **Predicted functions** | **Comments** | **References** |
| Phosphodies-terase | Rv0466 | Extracellular | Antimicrobial resistance | Similar to Rv2001 hypothetical 28.7 kDa protein from Mtb. Similarity to *M. bovis* Mb0475, *M. smegmatis* MSMEG_0909 | No information available |
|  | Rv0499 | Extracellular | Drug Resistance | Similarity to *M. bovis* Mb0510 and *M. smegmatis* MSMEG_0942 | Sun et al. (2012) |
|  | Rv0356c | Extracellular | No information available. | Equivalent to AL023514\|MLCB4_12 conserved hypothetical protein from *M. leprae* | No information available |
|  | Rv0163 | Extracellular | No information available. | Weak similarity with Rv2475c from Mtb | No information available |
|  | Rv2475c | Extracellular | No information available. | Some similarity with AAK44393\|Z97050\|MTCI28_3 conserved hypothetical protein from Mtb cosmid I | No information available |
|  | Rv1354c | Extracellular | Drug Resistance | Similar to Rv1357c (34.0% identity in 253 aa overlap) | Cui et al., (2009), and Segura-Cerd et al. (2018) |
|  | Rv1357c | Extracellular | Vaccination | Similar to Rv1354c\|(34.0% identity in 253 aa overlap) | Flores-Valdez et al. (2012) |
| Metallophos-phoesterase | Rv1571 | Extracellular | [Rna metabolic process](http://www.ebi.ac.uk/QuickGO/GTerm?id=Rna%20metabolic%20process) | Similar at N-terminal region to Q49625\|LEPB1170 _C3_227 hypothetical protein from *M. leprae* | No information available |
|  | Rv1670 | Extracellular | No information available. | Complete similarity to Mtb hypothetical proteins e.g. C-terminal region of Rv1056 | No information available |
|  | Rv3683 | Extracellular | No information available. | Equivalent to *M. bovis* Mb3708, *M. leprae* ML2309, *M. marinum* MMAR_5172, *M. smegmatis* MSMEG_6202 | No information available |
